# Research on Head-Mounted Virtual Reality and Computational Thinking Experiments to Improve the Learning Effect of AIoT Maker Course: Case of Earthquake Relief Scenes

**DOI:** 10.3389/fpsyg.2020.01164

**Published:** 2020-06-03

**Authors:** Shih-Yeh Chen, Ying-Hsun Lai, Yu-Shan Lin

**Affiliations:** ^1^Department of Computer Science and Information Engineering, National Taitung University, Taitung, Taiwan; ^2^Department of Information Science and Management System, National Taitung University, Taitung, Taiwan

**Keywords:** virtual reality, computational thinking, learning effect, Maker course, artificial internet of things

## Abstract

In this study, the head-mounted virtual reality (VR) technology is adpoted for computational thinking teaching in the AIoT Maker course teaching. The earthquake relief situation is designed in the VR in the course scenario, because in the context of situational thinking, pre-emptive training in the face of emergency disasters has been conducted through observation meetings or training courses. Through listening to lecturers or experienced personnel to share experiences, students often have a harder time thinking about real scenes and it is harder to think creatively how to design with the emergency disaster response. In view of this, this research will combine the development and evaluation of earthquake relief training courses for head-mounted VR and computational thinking experiments to explore the use of VR and computational thinking experiments to drive students to create ideas for real disaster relief scenarios. Through computational thinking, students think about different script situations and discuss in each scene to find a suitable maker design of the AIoT project. Finally, this study combined with its modular space program training to develop students’ programming skills. According to the experiment, this study is able to strength students’ practical learning motivation, and follow-up employ ability training for course learning.

## Introduction

Since the concept of Computational Thinking was put forward, experts and scholars in various fields have proposed different views of its definition, but most studies believe that it should include abstraction, composition, modeling and simulation, and algorithmic thinking (International Society for Technology in Education [ISTE] and Computer Science Teachers Association [CSTA], 2011; Mannila et al., 2014; Cetin, 2016). Therefore, computational thinking ability is not only the ability to use information tools, but the behavior to solve problems by using the thinking logic of information science. Many countries have taken how to cultivate students’ ability to solve practical problems by using computational thinking as one of the key directions of education. Due to the different cognitive abilities of students of different ages, the ability training methods, and teaching materials of information education should also be different (Guzdial, 2015; Philip, 2017); for example, in primary schools, students should be cultivated to use information application programs or try to experience logical judgment of program design, in order that they can learn simple information technology, and engage in the information experience. In high school, teachers can teach students basic information technology, guide them to engage with some basic concepts of calculator science, and train them to identify problems and design their thinking logic judgment to obtain solutions through algorithms. In this way, students can have more logical and structured thinking modes when learning other professional fields (Wiig and Wyly, 2016; Huang et al., 2019).

To sum up, this study combined head-mounted virtual reality (VR) and computational thinking experiments to improve students’ learning effectiveness in the IoT creation course, and took the earthquake relief scenarios as the training themes. In this way, students can use VR devices to experience earthquake disaster scenarios in the virtual world, consider how to disperse traffic and make the site accessible to ambulances and excavating equipment, or select a location to set up an on-site emergency command post. On one hand, the students keep abreast of the disaster situation and implement appropriate emergency response measures; on the other hand, they may ensure whether there is a nearby disaster and avoid the occurrence of secondary injuries. Finally, by identifying the physiological symptoms and degree of injury of the wounded, students may learn to identify the injury level of the wounded and transfer them to the hospital, etc.

Therefore, this study aimed to explore how to cultivate the computational thinking ability of students and enhance their learning performance through the head-mounted VR earthquake relief scenarios in the AIoT Creator Course. This study intended to explore students’ learning effectiveness in AIoT, in order to strengthen their learning experience. Through the training principle of paying equal attention to both theory and practice, this study cultivated students’ basic learning ability, and adopted the course strategy of computational thinking experiments to allow students to truly feel the practicability and importance of the basic disaster relief course knowledge and cultivate their own computational thinking ability. This study designed earthquake relief scenarios in head-mounted VR, and applied the basic principles of earthquake relief to such scenarios. In case of an earthquake disaster, disaster relief personnel will face changeable, urgent, and life-threatening situations, and if they cannot quickly grasp the status of the disaster site and the injuries of the wounded, there may be poor relief performance or casualties. Thus, basic disaster relief events were used to test students’ understanding of basic disaster relief knowledge and determine whether they can use the computational thinking mode to solve disaster relief events.

The remainder of this paper is organized, as follows. Section “Relevant researches” introduces the research related to computational thinking and VR technology in disaster relief teaching; section “Research method” proposes the method design; section “Results” analyzes and discusses the experimental data; section “Results” proposes the research contribution, limitation, and future research direction.

## Relevant Researches

### Computational Thinking

Computational thinking is a unique process for solving problems that reflects the basic thinking methods of information science. At present, computational thinking research can be roughly classified into two aspects: (1) connotations of computational thinking; (2) popularization and application of computational thinking; for example, the structural thinking research involved in the information discipline itself belongs to the connotation field of computational thinking. The application of computational thinking in various fields can be called generalized computational thinking. In March 2006, Professor Jeannette M. Wing, the Chairman of the Computer Science Department of Carnegie Mellon University, proposed and promoted computational thinking. She believed that computational thinking is a basic thinking method and skill, which is not limited to the research field of computer science, and everyone should learn and apply it in real life. In order to make it easier for people to understand computational thinking, Professor Wing further defined that computational thinking can reinterpret a seemingly complex problem into a method that can be solved by logical rules through abstraction, disassembly transformation, and simulation (Wing, 2006). Based on the research literature and the needs of curriculum development and evaluation in primary and secondary schools in China, Computing At School divided computational thinking into five abilities (abstraction, decomposition, algorithmic thinking, evaluation, and generalization) as the conceptual framework for developing computational thinking education (Jones and Humphreys, 2010):

1. Abstraction: A simplified process in which unnecessary details are deleted and only the key points are retained to make a problem simple and easy to understand.
2. Decomposition: Divide a complex problem into several small parts to make the problem easier to understand and consider.
3. Algorithmic thinking: Try to determine the rules and order of the issues to solve the problem and define them as clear steps: this step can be followed whenever a problem is encountered.
4. Evaluation: Judge whether a system or solution is effective, user-friendly, or qualified, and evaluate the advantages and disadvantages of each solution, etc.
5. Generalization: Determine the rules of the system, as well as the similarities and event links, as based on previous experience, and use them to solve similar problems.

Google’s Computational Thinking for Educators initially proposed 4 core capabilities for computational thinking (Google, 2018):

1. Decomposition: Decompose a task or problem into several steps or parts.
2. Pattern Recognition: Predict the pattern of the problem and determine the pattern for testing.
3. Abstraction: Determine the principles or factors that led to this pattern.
4. Algorithm Design: Design an instruction flow that can solve similar problems and be repeatedly executed.

With the popularization of computational thinking, Google defined computational thinking as a mental process and specific output required to solve problems by using computation, which may facilitate the development of computer applications and support problem solving in other disciplines, such as mathematics, science, engineering, and technology. In 2015, Google expanded the core capabilities of computational thinking from the original 4 items to 11 items:

1. Abstraction: Identify and extract relevant information to define key concepts.
2. Algorithm Design: Create an ordered set of instructions to solve similar problems or perform tasks.
3. System automation: Allow computers or machines to perform repetitive tasks.
4. Data collection: Collect data through the system.
5. Data analysis: Analyze data through search patterns or exploration methods.
6. Data representation: Describe and organize data with appropriate graphics, charts, text, or images.
7. Decomposition: Decompose data, processes, or problems into smaller, manageable parts.
8. Parallelization: Deal with various tasks at the same time to achieve common goals more effectively.
9. Pattern generalization: Create models, rules, principles, or theories that observe patterns to test predictive results.
10. Pattern recognition: Observe patterns, trends, and rules in data.
11. Simulation: Develop models to simulate real-world processes.

### VR Technology in Disaster Relief Teaching

Virtual reality is to display real environment scenery on a screen through the drawing function of a computer. As the depicted scenery has scene depth information and can be viewed and interacted with through VR devices, people will feel like it is a real scene. In fact, Stanley G. Weinbaum put forward the concept of VR in his novel *Pygmalion’s Spectacles* in 1935, and Morton Heilig realized the concept of VR in 1962 (Pan et al., 2006), thus, it can be seen that VR is not a new technology. However, due to the extremely high drawing capability requirements of VR, in the past, when computer operations were not yet mature, VR was just a display system in large research centers. As the software and hardware of information gradually matured, and the rise of mobile devices in recent years, the application field of VR can be expanded from the original indoor to outdoor (Innocenti et al., 2019). This has greatly expanded the application field of VR, including almost all fields that can use information technology, such as medical treatment, nursing, education, tourism, culture, military, architecture, design, engineering, scenic spot navigation, industry, and entertainment (Gavish et al., 2015). Some scholars recognized the user’s behavior and present the behavior in the VR environment to achieve the interaction between people and people or people and objects in the virtual space (Dai et al., 2019, 2020). Therefore, the application of VR technology in the field of medical care is increasing day by day. The medical care market of VR will reach USD 2.54 billion by 2020, mainly including disaster rescue, surgical application, medical rehabilitation, medical consultation, medical diagnosis, and medical education and training (Ammanuel et al., 2019; Hodgson et al., 2019).

## Research Method

**FIGURE 1:**
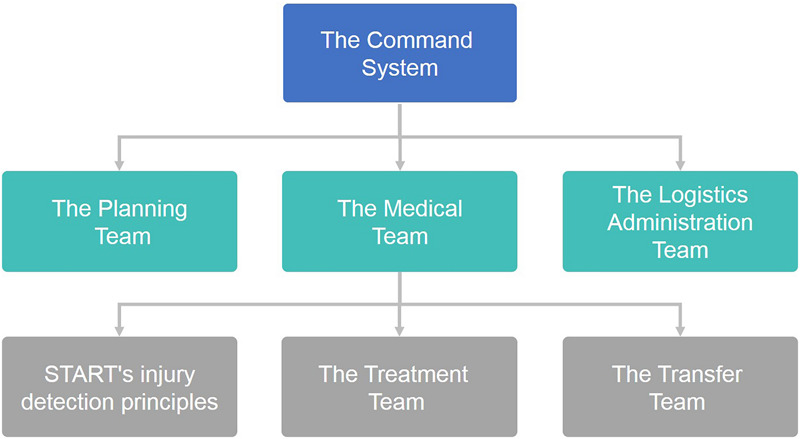
Head-mounted virtual reality and computational thinking course learning process.

This study integrated head-mounted VR into the development of earthquake relief courses, and the developed teaching materials were systematized and built into the AIoT creator course of the institute of technology, in order to facilitate the implementation and promotion of the curriculum in the future. This project intended to design and develop a computational thinking experiment for earthquake relief in a hospital in Tainan, and further revised and expanded it with a VR disaster relief course to record the participation of students in the course, in order to evaluate the influence of students’ thinking mode on computational thinking after completing an earthquake relief training course featuring a combination of VR and computational thinking experiments. Figure 1 shows the flow chart of this study.

### VR Disaster Relief Curriculum Development

First, this study focused on analyzing the requirements for the design of earthquake relief training scripts, and attempted to design VR courses as the basis of the subsequent development of the computational thinking experimental operation environment. In addition, doctors from a hospital in Tainan were invited to serve as experts for the development of the VR disaster relief courses, carry out joint literature review, and design the teaching materials. This study integrated the VR training courses into the earthquake disaster medical rescue courses, and sent it to experts for review. After reviewing the course materials and activities, this study developed the structure and function of the VR disaster relief course learning system, which served as the basis for the following computational thinking experiment operation and effectiveness evaluation. In this study, the development of the VR disaster relief training courses is divided into three items, namely:

1. Script design of earthquake disaster relief training courses.
2. Structure and content of VR earthquake relief training courses.
3. Design and development of computational thinking experiment.

#### Script Design of Earthquake Disaster Relief Training Courses

**FIGURE 2:**
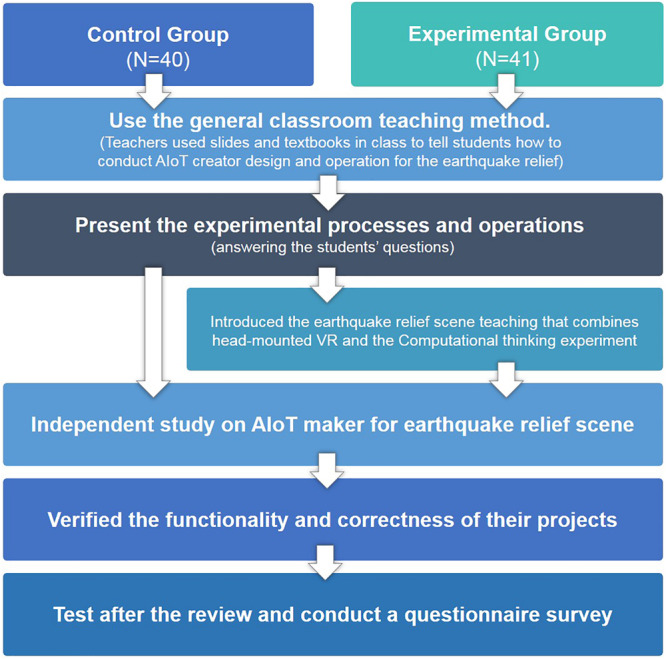
Basic organizational structure of the MOU.

In case of an earthquake disaster, the regional disaster medical rescue team will assign a medical operation unit (MOU) to carry out disaster relief tasks. The basic organizational structure of the MOU is shown in Figure 2. The command system is responsible for (1) establishing the command post, (2) assigning the main tasks of each department, (3) taking on the tasks that have not been assigned, (4) maintaining effective communication between each department, (5) correctly conveying information to the Ministry of Health and the media, and (6) deciding the termination of disaster medical rescue operations. The planning team is responsible for the following tasks: (1) carry out resource analysis, (2) collect relevant information, (3) analyze site conditions, and (4) brief the Commander at all times. The medical team is responsible for (1) classify patients according to START’s injury detection principles, (2) ask for appropriate additional manpower and medical support, (3) give proper treatment according to the severity of the patient’s classification, (4) transfer patients to appropriate medical institutions according to their medical needs, and (5) record the medical treatment and transfer data of patients. The logistics administration team is responsible for (1) providing and transferring necessary logistics supplies (including medical equipment, communication equipment, and living resources, etc.), (2) maintaining site safety, and (3) managing foreign rescue materials.

#### Structure and Content of VR Earthquake Relief Training Courses

Local health bureaus proposed to the Ministry of Health and Welfare that the local medical manpower was insufficient, and needed to be assisted by medical manpower across counties and cities. The Ministry of Health and Welfare launched the National Disaster Medical Assistance Team (NDMAT) to carry out humanitarian relief in disaster areas. The Emergency Operation Center (EOC) platform informs each medical team member to assemble on the platform, count the prepared items, and report to the designated place; assess the disaster area, select and set up emergency medical stations, and plan the area; determine the injuries of the wounded on site and report the injury data. The script is shown in Table 1.

**TABLE 1:** Disaster relief task design script.

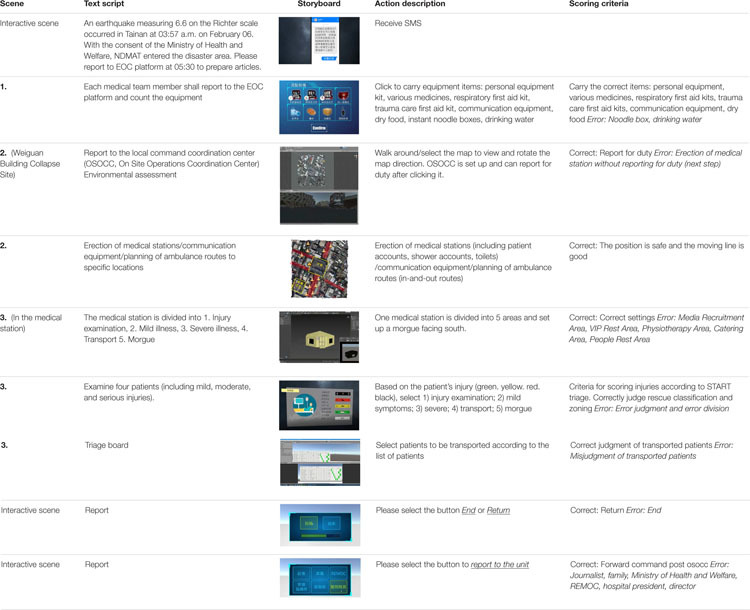

#### Design and Development of Computational Thinking Experiment

This plan is intended to carry out a complete computational thinking experimental design according to the virtual scene of disaster site reconstruction. No matter the direction of building collapse, the degree of building inclination, the degree of damage to surrounding houses, and the degree of road damage, etc., all the courses are designed according to the data of the day. Table 2 shows the computational thinking experiment of a disaster relief mission.

**TABLE 2:** Disaster relief mission computational thinking experiment.

	**Decomposition**	**Pattern recognition**	**Abstraction**	**Algorithm design**
Disaster relief center setup selection	1. How to get information of the earthquake scene 2. How to choose a location	1. Avoid being disturbed by aftershocks 2. Facilitate use by the wounded	Only consider the reverse direction and inclination angle of the earthquake building.	1. List selection location criteria 2. Check possible locations 3. Analyze the advantages and disadvantages of various points
Select disaster relief routes	1. How to choose the safest shortest path 2. The width of excavator, ambulance and truck should be met at the same time.	1. Facilitate use by the wounded 2. Facilitate the removal of gravel, bricks, and tiles.	Only consider the length of the path and the obstacles.	1. List all routes of rescue centers, collapsed buildings, and exits. 2. Check the size of obstacles on the route. 3. Choose possible paths and compare their advantages and disadvantages.
Classification and placement of the wounded	1. How to identify injury degree through vital signs 2. Number of wounded that can be treated by the medical team	1. Avoid misjudgment of classification 2. The number of wounded that can be accommodated and the degree of injuries that can be handled by hospitals in various regions	Quickly treating the wounded as the ultimate goal	1. Check the status of all the wounded 2. Design medical transport strategies for patients

### Flow of Course Teaching

**FIGURE 3:**
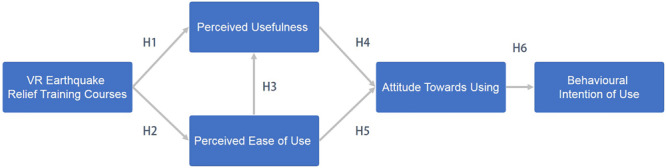
The AIoT Maker course experimental procedures.

The main purpose of this study is to discuss how to use different teaching strategies to explore the differences of learning effects between students with and without head-mounted VR and computational thinking experiments. The experimental flow is shown in Figure 3. The control group adopted the general classroom teaching method. Teachers used slides and textbooks in class to tell students how to conduct AIoT creator design and operation. The experimental group introduced the earthquake relief scene teaching that combines head-mounted VR and the computational thinking experiment. Teachers first offered the basic knowledge of earthquake relief, and then, guided students to practice how to carry out computational thinking experiments through VR, in order to learn to respond to earthquake relief. Then, teachers assigned the implementation topic of the creator course, asked the students in the two groups to carry out the AIoT creator project, and verified the functionality and correctness of their projects. After reminding students of their mistakes, teachers would give students the opportunity to practice again and determine whether students can correctly complete the implementation project. After the implementation project, teachers collected feedback from students and carried out a questionnaire survey according to students’ learning status, in order to determine whether students have increased learning effectiveness.

## Results

This study discussed the difference assessment of students’ experience of VR earthquake relief scenes and the evaluation of students’ learning effectiveness of the AIoT Creator Course after the computer thinking experiment.

### Experience Difference Evaluation of VR Earthquake Relief Scenes

In this study, the questionnaire survey method was used to investigate the impact of the introduction of VR technology on the students of earthquake relief training courses. The evaluation tool was mainly a learning impact questionnaire. The technology acceptance model (TAM; Davis, 1989) was used to design an experimental model to evaluate the impact, and to complete the evaluation results of this study together with the model.

#### Evaluation Model

**FIGURE 4:**
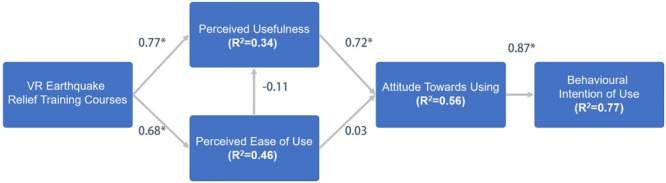
Research model of technology acceptance model.

Based on the theory of rational behavior, TAM inherited the basic spirit of rational behavior, which holds that belief perception will affect attitude, attitude will affect behavioral intention, and behavioral intention has obvious and positive influence on the actual use of the system. TAM put forward two factors in the acceptance degree of information system users, namely, perceived usefulness, and perceived ease of use. These two cognitive factors are regarded as people’s evaluation of performance and efforts, and the model is helpful for us to explore what factors affect the use of the system. Both perceived usefulness and perceived ease of use are affected by external variables, which refer to the influencing factors related to the system, or teaching mode. The evaluation model, as designed by this research method, is shown in Figure 4.

There are three external variables in the evaluation model designed by this research method, namely, VR characteristics, perceived cooperation, and perceived assistance, which also correspond to the VR implemented by this teaching method. These external variables will be detected by the evaluation model, and the eight hypotheses included in the model are described. VR courses provide a platform to enhance user experience (Franklin and Van Harmelen, 2007), as users can learn the professional knowledge of earthquake relief in the virtual world through a simple operational interface. By this method, users can deeply learn and feel what they have learned. Therefore, the first 2 hypotheses considered in the study are, as follows:

H1. The characteristics of VR are positively correlated with perceived usefulness—(H1)

H2. The characteristics of VR are positively correlated with perceived ease of use—(H2)

According to the theory of TAM, perceived ease of use and perceived usefulness are two factors that affect users’ attitude toward using new technologies, and perceived usefulness is also affected by the perceived ease of use. Then, the attitude of use will affect the intention of using new technologies. H3 – H6 are described, as follows:

H3. Perceived ease of use is positively correlated with perceived usefulness—(H3)

H4. Perceived ease of use is positively related to use attitude—(H4)

H5. Perceived usefulness is positively related to use attitude—(H5)

H6. Use attitude is positively related to use intention—(H6)

### Evaluation of Learning Effectiveness of Creator AIoT Maker Course

The main feature of creator teaching is to improve the verification of students’ learning results, which were determined by examination scores in the past. This study used a 360-degree evaluation method to evaluate students’ learning effectiveness, which can usually be extended outward by taking oneself as its center, thus, totaling 360 degrees. It can be divided into (1) vertical upward: teacher’s views of student performance (2) vertical downward: evaluator’s view of student performance (3) parallel to left: peer’s views of student performance (4) parallel to right: views of the same group members (5) oneself: students’ satisfaction with themselves. This study expected students to be evaluated through the four performance-oriented methods of 360-degree evaluation in each computational thinking experimental task; for example, when students want to learn the basic principles of AIoT, the teacher first explained the professional knowledge of AIoT, and gave application examples for real-life. After that, the teacher assigned AIoT creator tasks, asked students to start learning on their own, find possible solutions, and then, find and verify conclusions. When students encountered problems in class, the teacher encouraged them to find ways to determine solutions or gave tips as appropriate. Students may also ask their peers or team members to discuss possible directions.

## Results

The structured questionnaire for evaluating students was developed based on the characteristics of VR, sensory cooperation and sensory assistance, and TAMs. The students who filled in the questionnaires according to their degree of agreement with the description of the questionnaire items. The degree of agreement was divided into five levels, (1) representing strong agreement and (5) representing strongly disagree. The questionnaire model is presented in Table 3.

**TABLE 3:** Evaluation questionnaire.

**Evaluation questionnaire**	
**Evaluation variables**	**Question description**
(VC1)	This system provides an virtual environment for assigning creative tasks
(VC2)	This system provides a realistic environment for assigning creative tasks
(PE1)	I think this system is easy to use.
(PE2)	I think it’s quite easy to learn how to use this system.
(PE3)	I don’t think it takes much time to operate this system.
(PU1)	I think using this system will increase the efficiency of completing creative tasks.
(PU2)	I think using this system will improve the quality of creative tasks.
(PU3)	I think this system is helpful for creative tasks.
(AT1)	I like to use this system for creative tasks.
(AT2)	I can use this system with a positive attitude.
(AT3)	I think using this system is a good idea.
(IT1)	If I have the opportunity to use this system, I will use it for creative ideas.
(IT2)	If other courses use this system, I am willing to use it.

*VC, Virtual Reality Characteristics; PE, Perceived Ease-of-Use; PU, Perceived Usefulness; AT, Attitude Toward Using; and IT, Intent.*

In this study, we used measurement models and structural checks to evaluate. First, the measurement mode is used to evaluate aggregation validity, measurement reliability, and discriminant validity. Aggregate validity uses average variance extracted (AVE) that must exceed 0.5 of the standard minimum level. The reliability of the measurement mode is evaluated using combined reliability and Cronbach’s alpha. These values must be greater than the standard minimum level of 0.7. Discriminant validity is evaluated by comparing the correlation of potential variables with the square root of the average variation drawn. In this experiment, the correlation of all potential variables is less than the corresponding square root of the average variation extraction. The results of the measurement mode are shown in Table 4.

**TABLE 4:** Results of measurement mode.

	**Aggregation validity**	**Measure reliability**		**Discriminant validity**				
	**Average variation extraction**	**Combined reliability**	**Cronbach’s alpha**	**Latent variable correlation**				
				**WC**	**PU**	**PE**	**AT**	**IT**
VC	0.76	0.84	0.70	1.00				
PU	0.64	0.85	0.71	0.43	1.00			
PE	0.66	0.82	0.73	0.68	0.16	1.00		
AT	0.86	0.92	0.91	0.45	0.72	0.14	1.00	
IT	0.76	0.86	0.75	0.54	0.72	0.22	0.87	1.00

*VC, Virtual Reality Characteristics; PE, Perceived Ease-of-Use; PU, Perceived Usefulness; AT, Attitude Toward Using; and IT, Intent.*

**FIGURE 5:**
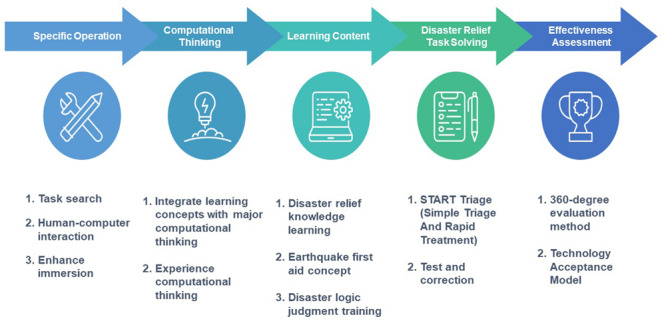
Structural pattern of technology acceptance model.

In the second part, the experiment uses a structural pattern to test the hypothetical path, which uses path coefficients and R2 values of TAM. As shown in Figure 5, this model shows that there is 35% variability in perceived usefulness, 45% variability in perceived ease-of-use, 55% variability in attitude toward using, and 78% in intention to use intent. From the results of the structural pattern, it is known that the two hypotheses H3 and H5 refute the predictions but other hypotheses are verified. The reason for refuting the hypotheses H3 and H5 should be that the experience of the VR earthquake relief scene is not a difficult task, because most of the participants are students from polytechnics. It makes operating different kinds of systems is simple and intuitive for them. Therefore, the perceived ease-of-use is not an obvious reason that can affect the perceived usefulness and attitude toward using. Overall, most of the hypotheses are confirmed in this study.

## Conclusion

In this study, an earthquake disaster relief training system is designed by combining VR with computational thinking. In the course, the teacher introduced the designed system through space design concept, scene science knowledge, algorithm data calculation, and engineering design configuration. Through the experiment course strategies of computational thinking, students can feel the practicality and importance of basic disaster relief curriculum knowledge and cultivate their own computational thinking ability. It is expected that students will be able to exert their creativity and thinking ability in a limited time through simulated emergency situations. In order to explore the effect of the proposed teaching strategies, the experimental method adopted a TAM and tested the model using the partial least squares method. The experimental results show that most of the hypotheses are verified, and the null hypothesis that the perceived ease-of-use is positively related to perceived usefulness and attitude toward using. The possible reason for the null hypothesis is that the participants are more familiar in system design, so the hypothesis “perceived ease-of-use” did not cause a positive correlation in the system usage. Although the proposed strategies in this study has validated the advantages of the system, the problem remains that we will explore the impact of perceived ease-of-use on students with science background. Another experiment will be designed to explore this reason in future work.

## Data Availability Statement

All datasets presented in this study are included in the article/supplementary material.

## Ethics Statement

Ethical review and approval was not required for the study on human participants in accordance with the local legislation and institutional requirements. Written informed consent from the patients was not required to participate in this study in accordance with the national legislation and the institutional requirements.

## Author Contributions

S-YC designed the flowchart and methodology, and analyzed the experimental results. Y-HL proposed this study conceptualization and designed the experimental environment. Y-SL formulated the research topic and manuscript structure, and polished grammar and sentence.

## Conflict of Interest

The authors declare that the research was conducted in the absence of any commercial or financial relationships that could be construed as a potential conflict of interest.
